# Optimal radiotherapy for patients with internal mammary lymph node metastasis from breast cancer

**DOI:** 10.1186/s13014-020-1464-0

**Published:** 2020-03-03

**Authors:** Kyungmi Yang, Haeyoung Kim, Doo Ho Choi, Won Park, Jae Myoung Noh, Won Kyung Cho

**Affiliations:** 1 Department of Radiation Oncology, Ajou University School of Medicine, Suwon, South Korea; 2 Department of Radiation Oncology, Samsung Medical Center, Sungkyunkwan University School of Medicine, 81, Irwon-ro, Gangnam-gu, Seoul, Republic of Korea 06351

**Keywords:** Breast cancer, Internal mammalian lymph node, Radiotherapy, Regional irradiation

## Abstract

**Background:**

This study aimed to determine the optimal radiotherapy (RT) regimen for patients with clinical metastasis to the internal mammary lymph node (cIMN+) from breast cancer.

**Methods:**

We retrospectively reviewed the medical records of 84 patients with cIMN+ breast cancer treated with curative surgery, taxane-based chemotherapy, and postoperative RT between January 2009 and December 2014. Postoperative RT was administered to the whole breast or chest wall using 50 Gy in 2 Gy fractions. Boost RT to the internal mammary lymph node (IMN) was administered at the physician’s discretion. We categorized patients into two groups according to the IMN dose as follows: low-dose IMN RT (50.0–63.5 Gy) and high-dose IMN RT (63.6–70.4 Gy).

**Results:**

After a median follow-up of 58 months (range, 12–111 months), IMN recurrence was observed in 2 patients (2.4%), and all IMN recurrences developed simultaneously with distant metastases. The 5-year locoregional recurrence-free survival, disease-free survival (DFS), and overall survival rates were 89.1, 72.0, and 81.2%, respectively. The triple-negative subtype, IMN size ≥1.0 cm, old age, and low-dose IMN were significantly associated with poor DFS. Among the patients with IMN size ≥1.0 cm, the 5-year DFS was significantly higher in those treated with high-dose IMN RT than in those treated with low-dose IMN RT (69.3% vs. 33.3%, *p* = 0.019).

**Conclusions:**

IMN RT without IMN dissection resulted in favorable outcomes in cIMN+ breast cancer. For patients with a large IMN, a higher IMN radiation dose might be needed for disease control.

## Introduction

Internal mammary node (IMN) involvement is a known poor prognostic factor for survival in patients with breast cancer. Patients with clinical IMN involvement (cIMN+) had a low survival rate with frequent distant metastasis compared to those without cIMN+ [1]. Previous surgical studies found that the frequency of pathologic IMN involvement was between 28 and 52% in patients with axillary lymph node (ALN) metastasis, while it was between 5 and 17% for patients without ALN metastasis [2, 3]. According to recent studies in which contemporary imaging modalities such as computed tomography (CT), magnetic resonance imaging (MRI) or positron emission tomography-computed tomography (PET-CT) were performed, the incidence of cIMN+ ranged between 11 and 16% in breast cancer patients with advanced nodal disease (cN2-N3) [4, 5]. Even though cIMN+ is frequently observed in patients with breast cancer, the optimal treatment method and prognosis have not been well identified.

In the past, radical mastectomy and IMN dissection were performed for patients with cIMN+, even though surgical treatment caused high morbidity without survival benefit [2]. In contrast, in recent years, multimodal treatments including breast surgery without IMN dissection, systemic therapy, and radiotherapy (RT) have been administered for patients with cIMN+ breast cancer. With these multimodal treatments, favorable outcomes could be achieved for cIMN+ breast cancer [4, 6]. To eradicate the tumor in the IMN without node dissection, high-dose RT is presumably necessary. However, the optimal radiation dose has not been determined for patients with IMN+ breast cancer.

Therefore, we performed this study to evaluate the outcomes of combined treatment including breast surgery, systemic treatment, and IMN-targeted RT. Through this analysis, we aimed to determine the prognostic significance of radiation dose in achieving disease control in patients with cIMN+ breast cancer.

## Methods and materials

We retrospectively reviewed medical records of patients who received postoperative RT for cIMN+ breast cancer at the Samsung Medical Center between January 2009 and December 2014. cIMN+ was defined as the IMN size of ≥0.5 cm on imaging studies at the time of breast cancer diagnosis. Imaging work-ups included chest CT, breast ultrasonography (US), breast MRI, or PET-CT. The size of the IMN was measured on breast MRI scans for all patients. Fine needle aspiration biopsy (FNABx) of the IMN was performed at the physician’s decision when IMN metastasis was uncertain on imaging studies. The inclusion criteria for this study were as follows: 1) newly diagnosed cIMN+ breast cancer, 2) patients receiving curative surgery, taxane-based chemotherapy, and postoperative RT, 3) completion of planned RT, and 4) having no distant organ metastases. A total of 84 patients with cIMN+ were included in this analysis. Patients were staged according to the 7th edition American Joint Committee on Cancer (AJCC) staging [7].

All patients underwent curative surgery including mastectomy or breast conserving surgery (BCS). Postoperative RT was administered to the whole breast or chest wall, supraclavicular lymph node (SCN), and the IMN, with a total dose of 50 Gy at 2 Gy per fraction. In patients who underwent BCS, a tumor bed boost was delivered after whole breast irradiation, using a total dose of 10–16 Gy at 2–3.5 Gy per fraction. Among 21 patients with SCN metastasis, an additional SCN dose of 6–15 Gy at 2–3 Gy per fraction was administered to 14 patients. Boost RT to the IMN was administered at the discretion of the attending physician, with a total dose of 6–16.5 Gy at 2–3.3 Gy per fraction. The total dose to the IMN was calculated using the biologically equivalent dose in 2 Gy fractions (EQD2) assuming the α/β ratio of 3.5 Gy [8]. Patients were categorized according to the EQD2 of the IMN chain as follows: 1) 50–63.5 Gy, low-dose IMN RT, and 2) ≥63.6 Gy, high-dose IMN RT by using the Recursive partitioning procedure in R 2.2.1 (R Development Core Team, Vienna, Austria, http://www.R-project.org). All patients underwent CT-simulation prior to RT. Three-dimensional conformal radiotherapy or intensity-modulated radiotherapy was performed in 82 patients and 2 patients, respectively. In radiotherapy planning, the bilateral lungs and heart were contoured as organs-at-risk (OAR). Dose-constraints for OAR were as follows: ipsilateral lung volume receiving 20 Gy or over < 30%, heart volume receiving 17 Gy or over < 10%, and mean heart dose of < 5 Gy.

Taxane-based chemotherapy was administered before or after surgery. Hormonal therapy or anti-human epidermal growth factor receptor type 2 (HER2) treatment was administered according to the tumor subtypes. Immunohistochemistry (IHC) of the breast tumors was performed for estrogen receptor (ER), progesterone receptor (PR), and HER2. Positivity of ER/PR was defined as an Allred score of 3 to 8. HER2 positivity was defined as staining of 3+ on IHC or 2+ on IHC along with positive results on fluorescence in situ hybridization or silver in situ hybridization.

The loco-regional recurrence-free survival (LRRFS), progression-free survival (PFS), and overall survival (OS) were calculated between the time of primary treatment initiation and the date of locoregional recurrence, cancer recurrence, and death, respectively. The severity of treatment-related toxicity was graded according to the Common Terminology Criteria for Adverse Events (CTCAE) version 4.0 [9]. Kaplan-Meier survival analysis was performed to estimate the survival rates, and the log-rank test was used to compare survival between groups with different variables. Univariate and multivariate analyses using Cox regression models were used to evaluate the influence of variables on survival. Variables with significance at *p* < 0.3 on univariate analysis were retained for multivariate analysis.

Values were considered statistically significant when *p* < 0.05. All statistical analyses were performed using SPSS Statistics version 22 (SPSS Inc., IBM, Chicago, IL, USA). This study was approved by the institutional review board of the Samsung Medical Center with No. 2019–02-081, and was classified exempt to obtain informed consent of the participants.

## Results

### Patient characteristics

Of 2114 patients who received postoperative radiotherapy for regional lymph node-positive breast cancer between 2009 and 2014, a total of 84 patients met the inclusion criteria for this study. The median age of patients was 41 years (range, 28–67 years). Most patients (92.9%) had invasive ductal carcinoma. All 84 patients had cIMN+ involving a single intercostal space (*n* = 37) or extending to multiple intercostal spaces (*n* = 47). Breast MRI was performed for all patients. Breast US, chest CT, and PET-CT were performed for 83 patients, 67 patients, and 72 patients, respectively. The median long diameter of the IMN was 0.9 cm (range, 0.5–1.8 cm). FNABx of the IMN was conducted for 43 patients at the time of breast cancer diagnosis. Among the 43 patients, 40 patients had a pathologically confirmed IMN metastasis.

Intensity-modulated RT was performed in 2 patients while conventional RT using partial wide tangent beams (*n* = 44) or photon-electron mixed fields (*n* = 38) were performed in 82 patients. Boost RT to the IMN region was applied for 69 patients after whole breast or chest wall irradiation. The median EQD2 to the IMN region was 63.6 Gy. A total of 35 patients (41.7%) received < 63.6 Gy to the IMN while 49 (58.5%) patients underwent ≥63.6 Gy EQD2 to the IMN chain. The patient characteristics are shown in Table 1. There was no significant difference in patient characteristics between the high-dose IMN RT group and the low-dose IMN RT group, except in the type of breast surgery (Table 2). More patients treated with mastectomy received high-dose radiation to the IMN compared to those treated with BCS (63.3% vs. 36.7%, *p* = 0.026).

**Table 1:** Patient characteristics

Characteristics		Number of patients (%)
Age (years)	≤40	38 (45.2%)
	> 40	46 (54.8%)
Laterality of breast cancer	Left breast	57 (67.9%)
	Right breast	27 (32.1%)
Location of breast cancer	Inner or center part	66 (78.6%)
	Outer part	18 (21.4%)
Histologic type	Invasive ductal carcinoma	78 (92.9%)
	Others	6 (7.1%)
Histologic grade	1–2	45 (53.6%)
	3	30 (35.7%)
	Unknown	9 (10.7%)
Tumor subtype	ER+/or PR+/HER2-	32 (38.1%)
	ER+/or PR+/HER2+	12 (14.3%)
	ER−/PR−/HER2+	10 (11.9%)
	ER−/PR−/HER2-	30 (35.7%)
cT stage	T1-T2	47 (56.0%)
	T3-T4	37 (44.0%)
cN stage	N2b or N3b	63 (75.0%)
	N3c	21 (25.0%)
Extent of IMN	Single ICS	37 (44.0%)
	Multiple ICS	47 (56.0%)
Long diameter of the IMN	< 1.0 cm	42 (50.0%)
	≥1.0 cm	42 (50.0%)
Type of breast surgery	Breast-conserving surgery	40 (47.6%)
	Mastectomy	44 (52.4%)
Type of axillary surgery	Sentinel lymph node biopsy	10 (11.9%)
	Axillary lymph node dissection	74 (88.1%)
Neoadjuvant chemotherapy	Performed	66 (78.6%)
	Not performed	18 (21.4%)
EQD2 of the IMN^1)^	50.0–63.5 Gy	35 (41.7%)
(median, 63.6 Gy; range, 50–70.4 Gy)	63.6–70.4 Gy	49 (58.3%)

^1)^Radiotherapy dose was calculated using the EQD2 assuming the α/β ratio of 3.5 Gy. *Abbreviations*: *IDC* Invasive ductal carcinoma; *ER* Estrogen receptor, *PR* Progesterone receptor, *HER2* Human epidermal growth factor receptor type 2, *ICS* Intercostal space, *IMN* Internal mammary node, *EQD2* Biologically equivalent dose in 2 Gy fractions

**Table 2:** Patient’s characteristics according to radiation dose to internal mammary lymph node

Characteristics		Number of patients (%)		*p*-value
		50–63.5 Gy ^1)^(*n* = 35)	63.6–70.4 Gy ^1)^(*n* = 49)	
Age	≤ 40 years	15 (42.9%)	23 (46.9%)	0.825
	> 40 years	20 (57.1%)	26 (53.1%)	
Histologic grade	1–2	23 (71.9%)	22 (51.2%)	0.096
	3	9 (28.1%)	21 (48.8%)	
Subtypes	Non-TNBC	22 (62.9%)	32 (65.3%)	0.822
	TNBC	13 (37.1%)	17 (34.7%)	
cT stages	1–2	19 (54.3%)	28 (57.1%)	0.827
	3–4	16 (45.7%)	21 (42.9%)	
cN stages	2b or 3b	26 (74.3%)	37 (75.5%)	1.000
	3c	9 (25.7%)	12 (24.5%)	
FNABx for IMN	(−) or unknown	20 (57.1%)	24 (49.0%)	0.511
	(+)	15 (42.9%)	25 (51.0%)	
Extent of the IMN	Single ICS	13 (37.1%)	24 (49.0%)	0.373
	Multiple ICS	22 (62.9%)	25 (51.0%)	
IMN long diameter	< 1.0 cm	20 (57.1%)	22 (44.9%)	0.376
	≥ 1.0 cm	15 (42.9%)	27 (55.1%)	
Neoadjuvant chemotherapy	Not done	9 (25.7%)	9 (18.4%)	0.433
	Done	26 (74.3%)	40 (81.6%)	
Primary surgery	BCS	22 (62.9%)	18 (36.7%)	0.026
	Mastectomy	13 (37.1%)	31 (63.3%)	
Axillary surgery	ALND	30 (85.7%)	44 (89.8%)	0.735
	SLNB	5 (14.3%)	5 (10.2%)	

^1)^Radiotherapy dose was calculated using the biologically equivalent dose in 2 Gy fractions (EQD2) assuming the α/β ratio of 3.5 Gy

Abbreviations: *TNBC* Triple-negative breast cancer, *IMN* Internal mammary node, *FNABx* Fine needle aspiration biopsy, *ICS* Intercostal space, *BCS* Breast conserving surgery, *ALND* Axillary lymph node dissection, *SLNB* Sentinel lymph node biopsy

### Patterns of failure, survival, and prognostic factors

After a median follow-up time of 58 months (range, 12–111 months), 15 patients died and 26 showed recurrence. The IMN recurrence was developed in 2 (2.4%) patients. All IMN recurrences were found simultaneously with distant metastases. One of the IMN recurrences was found in a patient who received 66 Gy of IMN irradiation, at 6 months after the completion of RT. The other IMN recurrence was noted after 16 months after the completion of RT in a patient who received 50 Gy of IMN RT. The sites of the first recurrence were as follows: loco-regional recurrence only, 2 patients; distant metastasis only, 15 patients; and simultaneous locoregional and distant recurrences, 9 patients. Among the 24 patients with distant metastases, the metastatic sites were as follows: non-regional lymph nodes only, 3 patients; visceral organs only, 6 patients; bone only, 6 patients; and simultaneous multi-organ involvement, 9 patients.

**Fig. 1:**
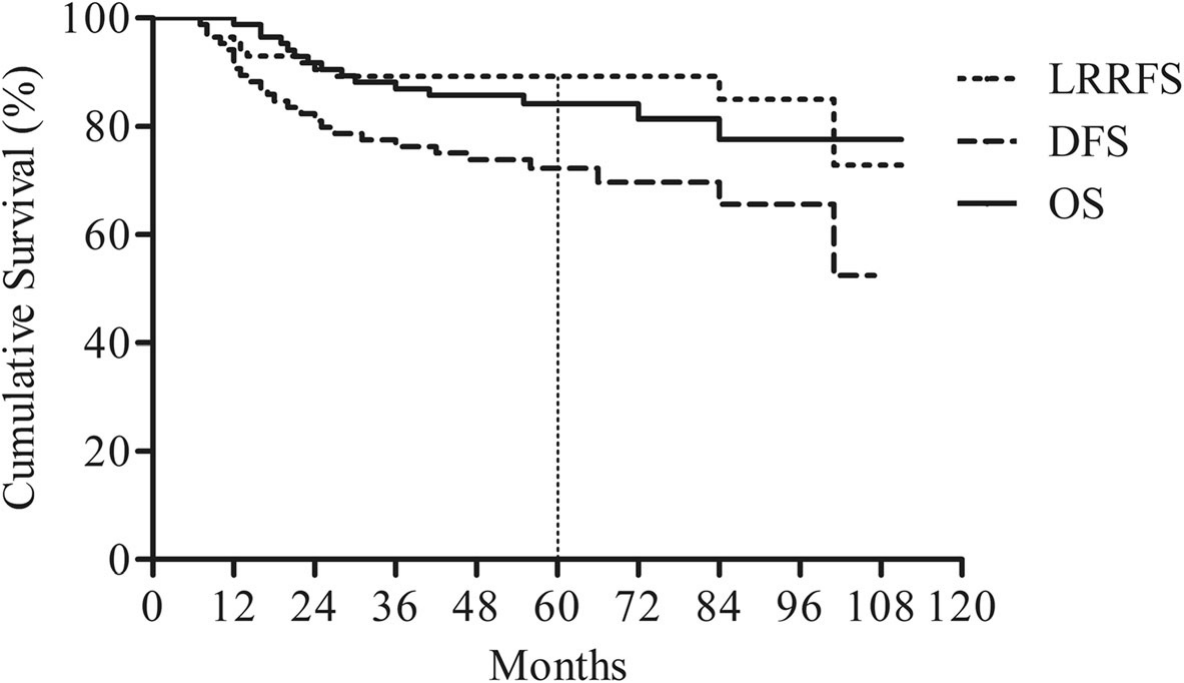
Clinical outcomes of breast cancer patients with clinically positive IMN. *Abbreviations*: IMN, internal mammary node, LRRFS, Loco-Regional Recurrence-Free Survival; DFS, Disease-Free Survival; OS, Overall Survival

**Fig. 2:**
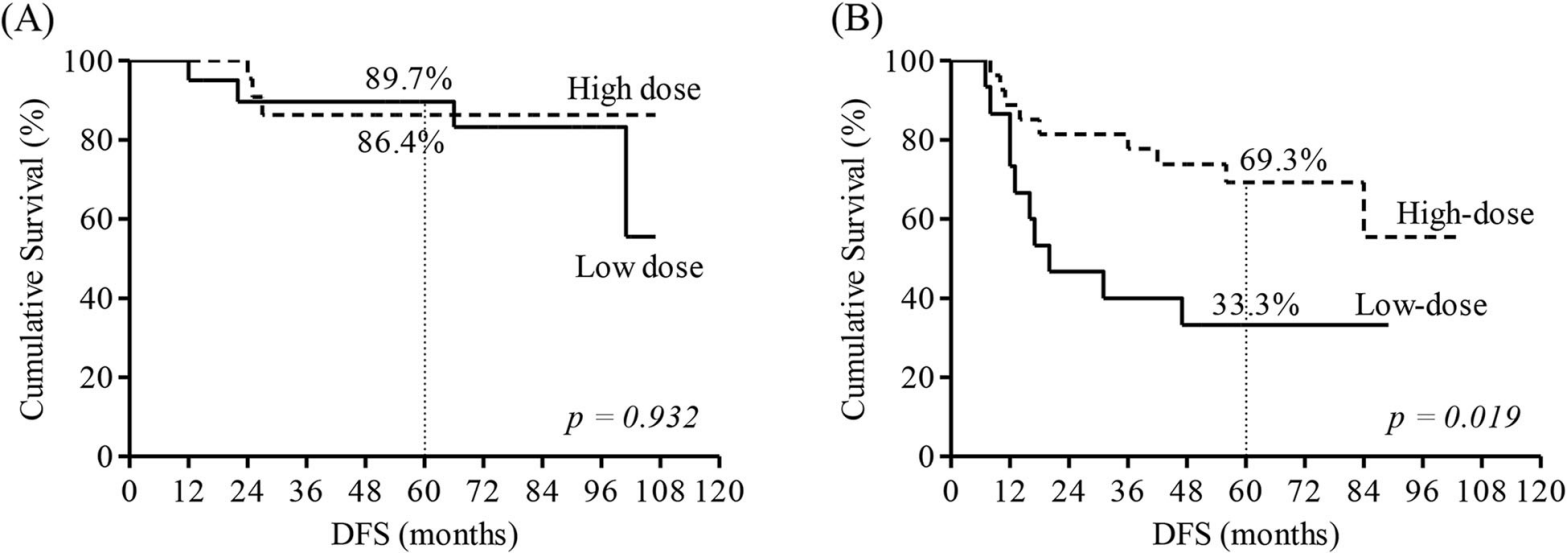
Disease-free survival according to the internal mammary lymph node size and radiation dose^*^ to the internal mammary lymph node (**a**) In patients with the internal mammary lymph node size < 1.0 cm (*n* = 42). (**b**) In patients with the internal mammary lymph node size ≥1.0 cm (*n* = 42). ^*^Radiation dose to the IMN was grouped as follows: 50 Gy–63.5 Gy, low-dose; and ≥ 63.6 Gy, high-dose

The 5-year LRRFS, DFS, and OS rates were 89.1, 72.0, and 81.2%, respectively (Fig. 1). Among the clinical factors, patient age, tumor subtype, size of the IMN, and radiation dose to the IMN were significantly associated with DFS. Age >  40 years, triple-negative breast cancer (TNBC) subtype, IMN size ≥1.0 cm, and receiving IMN radiation dose < 63.6 Gy were significantly associated with inferior DFS (Table 3). We compared patient DFS depending on the IMN size and IMN radiation dose. The patient characteristics were not significantly different depending on the size of the IMN (Table 4). The IMN radiation dose had a different prognostic significance depending on the IMN size. In patients with IMN size ≥1.0 cm, high-dose IMN RT was significantly associated with better DFS compared to low-dose IMN RT (69.3% vs. 33.3%, *p* = 0.019, Fig. 2b). However, in patients with IMN size < 1.0 cm, DFS was not influenced by the IMN radiation dose (Table 5, Fig. 2a). TNBC subtype was significantly associated with worse LRRFS in comparison to non-TNBC tumors (Table 6). Old age and TNBC subtype were significant factors linked to worse OS (Table 7).

**Table 3:** Prognostic factors of disease-free survival

Characteristics		5-year DFS (%)	Univariate *p*-value	Multivariate *p*-value	HR (95% CI)
Age	≤40 years (n = 38)	85.5%	0.007	0.012	0.301 (0.118–0.767)
	> 40 years (*n* = 46)	60.7%			
Tumor subtype^1)^	Non-TNBC (*n* = 54)	80.7%	0.012	< 0.001	0.189 (0.079–0.453)
	TNBC (*n* = 30)	56.7%			
cT stages	1–2 (*n* = 47)	77.9%	0.090	0.070	0.442 (0.183–1.068)
	3–4 (*n* = 37)	64.8%			
cN stages	2b or 3b (*n* = 63)	72.1%	0.576	–	–
	3c (*n* = 21)	71.4%			
Malignant cells on FNABx of the IMN	(−) or unknown (*n* = 44)	73.7%	0.610	–	–
	(+) (*n* = 40)	65.4%			
Extent of IMN	Single ICS (*n* = 37)	74.9%	0.525	–	–
	Multiple ICS (*n* = 47)	63.4%			
IMN long diameter	< 1.0 cm (*n* = 42)	87.9%	0.002	< 0.001	0.157 (0.060–0.412)
	≥1.0 cm (*n* = 42)	56.4%			
Neoadjuvant chemotherapy	Performed (*n* = 66)	72.1%	0.927	–	–
	Not performed (*n* = 18)	71.1%			
Axillary surgery	ALND (*n* = 74)	69.5%	0.184	0.056	8.003 (0.947–67.663)
	SLNB (*n* = 10)	90.0%			
EQD2 of the IMN^2)^	50.0–63.5 Gy (*n* = 35)	65.1%	0.188	0.029	2.491 (1.095–5.663)
	63.6–70.4 Gy (*n* = 49)	76.6%			

^1)^TNBC was defined as tumors that were negative for the estrogen receptor, progesterone receptor, and human epidermal growth factor receptor 2 on immunohistochemical staining of the breast tumor

^2)^Radiotherapy dose was calculated using the EQD2 assuming the α/β ratio of 3.5 Gy

Abbreviations: *DFS* Disease-free survival, *TNBC* Triple-negative breast cancer, *FNABx*, fine needle aspiration biopsy, *IMN* Internal mammary node, *ICS* Intercostal space, *ALND* Axillary lymph node dissection, *SLNB* Sentinel lymph node biopsy; *HR* Hazard ratio; *CI* Confidence interval, *EQD2* Biologically equivalent dose in 2 Gy fractions

**Table 4:** Comparisons between patients with small internal mammary lymph nodes (< 1.0 cm) and patients with large internal mammary lymph node (≥1.0 cm)

Characteristics		Number of patients (%)		*p*-value
		IMN size < 1.0 cm,(*n* = 42)	IMN size ≥1.0 cm,(*n* = 42)	
Age	≤40 years	20 (47.6%)	18 (42.9%)	0.827
	> 40 years	22 (52.4%)	24 (57.1%)	
Histologic grade	1–2	23 (54.8%)	22 (52.4%)	0.638
	3	13 (31.0%)	17 (40.5%)	
Subtypes^1)^	Non-TNBC	29 (69.0%)	25 (59.5%)	0.495
	TNBC	13 (31.0%)	17 (40.5%)	
cT stages	1–2	23 (54.8%)	24 (57.1%)	1.000
	3–4	19 (45.2%)	18 (42.9%)	
cN stages	2b or 3b	30 (71.4%)	33 (78.6%)	0.615
	3c	12 (28.6%)	9 (21.4%)	
Malignant cells on FNABx of the IMN	(−) or unknown	22 (52.4%)	22 (52.4%)	1.000
	(+)	20 (47.6%)	20 (47.6%)	
Extent of IMN	Single ICS	20 (47.6%)	17 (40.5%)	0.661
	Multiple ICS	22 (52.4%)	25 (59.5%)	
Neoadjuvant chemotherapy	Performed	29 (69.0%)	37 (88.1%)	0.061
	Not performed	13 (31.0%)	5 (11.9%)	
Primary surgery	BCS	20 (47.6%)	20 (47.6%)	1.000
	Mastectomy	22 (52.4%)	22 (52.4%)	
Axillary surgery	ALND	39 (92.9%)	35 (83.3%)	0.313
	SLNB	3 (7.1%)	7 (16.7%)	
EQD2 of the IMN^2)^	50.0–63.5 Gy	20 (47.6%)	15 (35.7%)	0.376
	63.6–70.4 Gy	22 (52.4%)	27 (64.3%)	

^1)^TNBC was defined as tumors that were negative for estrogen receptor, progesterone receptor, and human epidermal growth factor receptor 2 on immunohistochemical staining of the breast tumor

^2)^Radiotherapy dose was calculated using the EQD2 assuming the α/β ratio of 3.5 Gy

Abbreviations: *IMN* Internal mammary node; *TNBC* Triple-negative breast cancer, *FNABx* Fine needle aspiration biopsy, *ICS* Intercostal space, *BCS* Breast conserving surgery, *ALND* Axillary lymph node dissection, *SLNB* Sentinel lymph node biopsy, *EQD2* Biologically equivalent dose in 2 Gy fractions

**Table 5:** Disease-free survival according to the size of the internal mammary lymph node

Characteristics		IMN size < 1.0 cm (n = 42)		IMN size ≥1.0 cm (n = 42)	
		5-year DFS	*p*-value	5-year DFS	*p*-value
Age	≤40 years	100.0%	0.009	71.3%	0.131
	> 40 years	77.3%		45.1%	
Subtypes^1)^	Non-TNBC	96.4%	0.031	63.5%	0.120
	TNBC	69.2%		47.1%	
cT stages	1–2	91.1%	0.267	65.2%	0.119
	3–4	84.2%		44.4%	
cN stages	2b or 3b	89.7%	0.118	56.4%	0.847
	3c	83.3%		55.6%	
Malignant cells on FNABx of the IMN	(−) or	86.5%	0.327	54.5%	0.930
	unknown (+)	89.7%		58.7%	
Extent of IMN	Single ICS	90.0%	0.899	57.0%	0.658
	Multiple ICS	85.9%		56.0%	
Neoadjuvant chemotherapy	Not performed	83.3%	0.852	40.0%	0.417
	Performed	89.7%		58.5%	
EQD2 of the IMN^2)^	50.0–63.5 Gy	89.7%	0.932	33.3%	0.019
	63.6–70.4 Gy	86.4%		69.3%	

^1)^TNBC was defined as tumors that were negative for estrogen receptor, progesterone receptor, and human epidermal growth factor receptor 2 on immunohistochemical staining of the breast tumor

^2)^Radiotherapy dose was calculated using the EQD2 assuming the α/β ratio of 3.5 Gy

Abbreviations: *IMN* Internal mammary node, *DFS* Disease-free survival, *TNBC* Triple-negative breast cancer, *FNABx* Fine needle aspiration biopsy, *ICS* Intercostal space, *EQD2* Biologically equivalent dose in 2 Gy fractions

**Table 6:** Prognostic factors of locoregional-recurrence free survival

Characteristics		5-year LRRFS	Univariate *p*-value	Multivariate *p*-value	HR (95% CI)
Age	≤40 years (*n* = 38)	94.7%	0.184	0.187	0.397(0.010–1.564)
	> 40 years (*n* = 46)	84.3%			
Tumor subtype^1)^	Non-TNBC (*n* = 54)	96.3%	0.009	0.004	0.120(0.029–0.500)
	TNBC (*n* = 30)	75.8%			
cT stages	1–2 (*n* = 47)	87.1%	0.568	–	–
	3–4 (*n* = 37)	91.7%			
cN stages	2b or 3b (*n* = 63)	88.7%	0.915	–	–
	3c (*n* = 21)	90.2%			
Malignant cells on FNABx of the IMN	(−) or unknown (*n* = 44)	89.8%	0.961	–	–
	(+) (*n* = 40)	88.5%			
Extent of IMN	Single ICS (*n* = 37)	89.0%	0.660	–	–
	Multiple ICS (*n* = 47)	89.2%			
IMN long diameter	< 1.0 cm (*n* = 42)	92.5%	0.267	0.129	0.365(0.099–1.330)
	≥1.0 cm (*n* = 42)	85.7%			
Neoadjuvant chemotherapy	Performed (*n* = 66)	92.3%	0.108	0.167	0.242(0.034–1.626)
	Not performed (*n* = 18)	77.0%			
Axillary surgery	ALND (*n* = 74)	88.9%	0.889	–	–
	SLNB (*n* = 10)	90.0%			
EQD2 of the IMN^2)^	50.0–63.5 Gy (*n* = 35)	85.5%	0.544	–	–
	63.6–70.4 Gy (*n* = 49)	91.7%			

^1)^TNBC was defined as tumors that were negative for the estrogen receptor, progesterone receptor, and human epidermal growth factor receptor 2 on immunohistochemical staining of the breast tumor

^2)^Radiotherapy dose was calculated using the EQD2 assuming the α/β ratio of 3.5 Gy

Abbreviations: *LRRFS* Locoregional-recurrence free survival, *TNBC* Triple-negative breast cancer; *FNABx* Fine needle aspiration biopsy, *IMN* Internal mammary node, *ICS* Intercostal space, *ALND* Axillary lymph node dissection; *SLNB* Sentinel lymph node biopsy, *HR* Hazard ratio, *CI* confidence interval, *EQD2* Biologically equivalent dose in 2 Gy fractions

**Table 7:** Prognostic factors of overall survival

Characteristics		5-year OS	Univariate *p*-value	Multivariate *p*-value	HR (95% CI)
Age	≤40 years (*n* = 38)	94.6%	0.007	0.009	0.132(0.029–0.609)
	> 40 years (n = 46)	75.4%			
Tumor subtype^1)^	Non-TNBC (n = 54)	92.0%	0.016	0.003	0.167(0.051–0.548)
	TNBC (n = 30)	70.0%			
cT stages	1–2 (n = 47)	87.0%	0.232	0.067	3.049(0.925–10.054)
	3–4 (n = 37)	80.1%			
cN stages	2b or 3b (n = 63)	83.3%	0.492	–	–
	3c (*n* = 21)	85.7%			
Malignant cells on FNABx of the IMN	(−) or unknown (*n* = 44)	84.5%	0.557	–	–
	(+) (n = 40)	83.4%			
Extent of IMN	Single ICS (*n* = 37)	89.1%	0.045	0.845	0.890(0.275–2.880)
	Multiple ICS (*n* = 47)	79.9%			
IMN long diameter	< 1.0 cm (*n* = 42)	92.7%	0.053	0.052	1.621(0.318–8.255)
	≥1.0 cm (*n* = 42)	75.7%			
Neoadjuvant chemotherapy	Performed (*n* = 66)	82.9%	0.493	–	–
	Not performed (*n* = 18)	87.8%			
Axillary surgery	ALND (*n* = 74)	83.2%	0.556	–	–
	SLNB (*n* = 10)	90.0%			
EQD2 of the IMN^2)^	50.0–63.5 Gy (*n* = 35)	85.3%	0.926	–	–
	63.6–70.4 Gy (*n* = 49)	82.8%			

^1)^TNBC was defined as tumors that were negative for the estrogen receptor, progesterone receptor, and human epidermal growth factor receptor 2 on immunohistochemical staining of the breast tumor

^2)^Radiotherapy dose was calculated using the EQD2 assuming the α/β ratio of 3.5 Gy

Abbreviations: *OS* Overall survival, *TNBC* Triple-negative breast cancer, *FNABx* Fine needle aspiration biopsy, *IMN* Internal mammary node, *ICS* Intercostal space, *ALND* Axillary lymph node dissection, *SLNB* Sentinel lymph node biopsy, *HR* Hazard ratio, *CI* Confidence interval, *EQD2* Biologically equivalent dose in 2 Gy fractions

### Treatment-related toxicity

There were no cases of grade ≥ 3 toxicity. Grade 2 toxicity was found in 6 patients: dermatitis in 4 patients, pneumonitis in 1 patient, and cardiac disorder in 1 patient. Cardiac toxicity, presenting as diastolic dysfunction, was found 5 years after the completion of RT in a patient with left breast cancer**.** The patient had received 50 Gy of RT to the left chest wall, SCN, and IMN region. Medication for diastolic dysfunction was continued for 7 months. At the time of data collection for this study, the patient had no symptoms associated with the cardiac disease after medication.

## Discussion

In patients with cIMN+ breast cancer, a combination of breast surgery, postoperative RT, and taxane-based chemotherapy resulted in favorable outcomes even without IMN dissection. Less than 3% of our patients had IMN recurrence after the combination treatment. Old age, TNBC, and large IMN were associated with poor DFS. In patients with a large IMN, the IMN radiation dose significantly affected the DFS. The EQD2 > 63.5 Gy was associated with improved DFS in patients with IMN size ≥1.0 cm. However, the association between IMN radiation dose and DFS was not significant in patients with IMN size < 1.0 cm. Therefore, it might be necessary to modify the IMN radiation dose according to the size of the IMN during postoperative RT for cIMN+ breast cancer.

The IMN is situated in the parasternal region surrounded by the interpectoral muscle, fibrofatty tissue, and the internal mammary vessels [10]. As the IMN is located in narrow intercostal spaces adjacent to the internal mammary vessels, it is difficult to perform a biopsy the IMN. Even at the time of breast surgery, a separate incision might be needed to excise the IMN when BCS is performed for laterally located breast cancer [11]. According to a study, approximately 40% of patients with cIMN+, diagnosed through imaging studies, had negative results on FNABx [12]. Over 80% of the FNABx-negativity was caused by sample inadequacy or poor visibility of the IMN. Likewise, pathologic confirmation of IMN status is not always feasible when IMN adenopathy is observed on imaging studies. Previous studies showed that the rate of pathologic confirmation of IMN metastasis ranged between 9 and 57% in patients with cIMN+ breast cancer [4, 12, 13]. Given the difficulty in performing biopsy of the IMN, a diagnosis of IMN metastases is made based on radiologic findings in many clinical situations. Therefore, it is necessary to consider the radiologic features of the IMN in optimizing the management of cIMN+ breast cancer. Moreover, the RT regimen is needed to be modified according to characteristics of the IMN.

In previous studies, an IMN diameter of 0.5 cm or larger in breast MRI has been regarded as metastatic IMN [4, 14, 15]. In a study where MRI findings of IMN were compared with surgically dissected IMN, the IMN of ≥0.5 cm was likely to have malignant cells in pathologic examination with 90.7% accuracy, 93.3% sensitivity, and 89.3% specificity [15]. Similarly, in a study where IMN metastasis was determined based on pathologic evaluation or PET-CT finding, IMN short-axis length ≥ 0.4 cm was predictive of positive metastasis with 92.5% sensitivity and 84.2% specificity [14]. According to recent studies of physiologic IMN adenopathy incidentally found in healthy females undergoing screening breast MRI, the mean IMN diameter was 0.45 cm [16] or 0.4 cm [17]. Based on above-mentioned studies, we defined IMN ≥ 0.5 cm on imaging studies as clinically positive for metastasis in our study. Considering that the size criterion for positive metastasis is generally regarded as 0.9–1.0 cm for axillary lymph node [18, 19], the size criterion of IMN metastasis is thought to be smaller than that of axillary lymph node metastasis.

IMN dissection had been used for the treatment of cIMN+ breast cancer. In a randomized controlled trial comparing the outcome between radical mastectomy and extended radical mastectomy including IMN dissection, locoregional control was better with IMN dissection. However, patient’s overall survival and disease-free survival were not affected by the addition of IMN dissection [2]. Furthermore, other trials conducted before the 1980s reported that IMN dissection was not associated with improved survival in patients with breast cancer [20, 21]. To perform IMN dissection, additional skin incisions and chest tube placement might be necessary. In addition, division of intercostal muscle and transection of the ribs are also needed during surgery for IMN dissection [11]. Given the absence of a survival benefit and the possibility of surgical morbidity, surgical dissection of the IMN was abandoned [20, 21]. Currently, a combination of breast surgery, systemic therapy, and RT encompassing the IMN is a common approach for treating cIMN+ breast cancer. With the introduction of effective systemic therapy such as chemotherapy, anti-hormonal therapy, or targeted therapy, the oncologic outcomes have improved in patients with breast cancer [22, 23]. In addition, advanced RT techniques enable precise targeting of the tumor, thereby allowing sufficient irradiation of the IMN region [24]. Such advances in the treatment of breast cancer have resulted in favorable tumor control in patients with cIMN+ breast cancer. There have been studies that reported the outcomes of multimodal treatments without IMN dissection for cIMN+ breast cancer (Table 8) [4, 12, 25–28]. The IMN control rate was excellent, with an IMN recurrence rate of 0–11% after multimodal treatments. The 5-year DFS rate was 56–72% in the studies. Similarly, in our study, we found excellent IMN control (crude rate of 97.6%) and favorable DFS (72% at 5 years) after combined modality treatment for cIMN+ breast cancer. Therefore, it is more appropriate to administer a combination treatment including IMN-targeting RT and systemic therapy rather than IMN dissection for patients with cIMN+ breast cancer.

**Table 8:** Summary of studies in which multimodal treatment was performed without dissection of the internal mammary lymph node for patients with breast cancer and internal mammary lymph node metastases

Authors	No. of patients	Median FU (months)	Pathologic confirmation of IMN+	Chemotherapy regimen	Median IMN RT dose, (range)	IMN recurrence	5-year survival rates
Zhang et al. (4)	96	41	9%	AT-based (100%)	60.0 Gy (50.0–72.0 Gy)	11%	DFS 56%, OS 76%
Park et al. (25)^1)^	15	38	0%	T-based (73%), A-based (20%)	50.4 Gy (50.4–55.8 Gy)	6.7%	DFS 67%, OS 79%
Noh et al. (26)^1)^	45	57	40%	AT (54.5%), AC (29.1%)	50.0–50.4 Gy +/− boost	0%	DFS 66%, OS 76%
Joo et al. (12)	70	51	57%	T-based (94%)	60.0 Gy (56.0–66.0 Gy)	2.9%	DFS 72%, OS 77%
Sachdev et al. (27)	25	38	Not reported	Not reported	50.4 Gy (45.0–64.4 Gy)	0%	Not reported
Kim et al. (28)	95	43	2%	Not reported	50.0 Gy +/− boost (*n* = 12)	3.2%	DFS 70%, OS 84%
The present study	84	58	48%	T-based (100%)	62.5 Gy (50.0–66.5 Gy)	2.4%	DFS 72%, OS 81%

^1)^The studies included patients with internal mammary lymph node or supraclavicular lymph node metastasis from breast cancer

Abbreviations *FU* Follow-up, *IMN+* Metastasis to the internal mammary lymph node, *IMN* Internal mammary node, *RT* Radiotherapy, *A* Adriamycini, *T* Taxane, *DFS* Disease-free survival, *OS* Overall survival

IMN-targeting RT is essential for treating cIMN+ breast cancer; however, there have been few studies evaluating optimal radiation dose for cIMN+ breast cancer. A radiation dose of 45–50 Gy to the whole breast or chest wall plus a radiation boost to gross lesions has been recommended as a general guideline for postoperative RT for breast cancer [29]. For eradicating the gross tumor in the IMN, boost irradiation with 6–16 Gy has been administered to the IMN region in previous studies [4, 12, 25–28]. Accordingly, the median radiation dose to the IMN was 50.0–63.6 Gy in the previous studies. In our study, the median IMN dose was slightly higher compared to that in other studies. Moreover, a higher IMN radiation dose tended to be administered to patients with IMN size ≥1.0 cm compared to those with IMN size < 1.0 cm. These RT regimens are probably associated with a favorable IMN control in our study.

We noted a significant influence of the IMN dose on DFS in the current study. A higher IMN dose was closely associated with better DFS. Nonetheless, the dose-response effect was evident only in patients with IMN size ≥1.0 cm and not in those with IMN size < 1.0 cm. The 5-year DFS in patients with IMN size < 1.0 cm was high (86.4–89.7%), irrespective of the IMN RT dose. However, among the patients with IMN size ≥1.0 cm, the 5-year DFS rate was only 33.3% after low-dose IMN RT while it was 69.3% after high-dose IMN RT. The difference in the influence of the radiation dose depending on the IMN size might be owing to the IMN tumor burden. In patients with the IMN < 1.0 cm, there’s thought to be small IMN tumor burden, which can be eradicated with moderate dose RT and systemic treatments. On the contrary, patients with the IMN ≥1.0 cm might have large IMN tumor burden, which is resistant to moderate dose radiation and contemporary systemic agents. Ineffective control of IMN metastasis might allow spreading of cancer cells to distant organs, thereby resulting in poor DFS. In the meantime, it is probable that the above-mentioned dose-response relationship was resulted from an inequivalent distribution of pathological IMN metastasis between the patients with IMN < 1.0 cm and those with IMN ≥1.0 cm. Even though the number of patients with a positive FNABx was not different between the patients with IMN < 1.0 cm and those with IMN ≥1.0 cm, half of our patients did not have pathologic confirmation of IMN metastasis. Further studies are necessary to accurately interpret the dose-response effect found in the current analysis. The optimal cutoff value of the EQD2 to the IMN was 63.6 Gy in our study. Given that the 5-year DFS rate was 69.3% in patients with IMN size ≥1.0 cm after EQD2 of 63.6–70.4 Gy, there is still room for further improvement. More intensified treatment strategies—such as applying higher IMN radiation doses or administering more effective systemic agents—might be required to achieve better outcomes for patients with large IMN metastasis. In addition, modifying the IMN radiation dose according to the response after neoadjuvant chemotherapy (NAC) can help in optimizing the RT dose for cIMN+ breast cancer [4, 30]. Further studies are necessary to determine the optimal RT regimen for patients with cIMN+ breast cancer.

In our study, we found that patients 40 years old or younger had superior DFS than those of over 40 years of age. Contrary to our finding, previous studies showed that young age is an adverse prognostic factor for survival in patients with breast cancer [31, 32]. Breast cancer in young patients is likely to have more aggressive biological features such as high grade or triple-negative subtype as compared to those arising in older patients [31]. In a recent study, young age was significantly associated with increased risk of breast cancer death, but only among patients with luminal type tumor. The negative prognostic effect of young age was not found among patients with TNBC or HER2+ tumor [32]. Likewise, it seems that a worse breast cancer outcome in young patients is linked to the tumor characteristics, not the young age per se. In our study, young patients had a better breast cancer outcome than older patients. Given that tumor characteristics were not different between the age groups in our study (Table 9), other factors might contribute to the worse outcome in the patients > 40 years old. In previous studies on cIMN+ breast cancer, the prognostic impact of the patient’s age on breast cancer outcome has not been evaluated [4, 12, 25, 26, 28]. Further studies are necessary to determine whether age is significantly associated with survival in patients with cIMN+ breast cancer.

**Table 9:** Patient’s characteristics according to age groups

Characteristics		Number of patients (%)		*p*-value
		≤ 40 years (*n* = 38)	> 40 years(*n* = 46)	
Follow-up duration	Median months (range)	58.5 mo (15–111)	59.0 mo (12–104)	0.215 ^1)^
Histologic grade	1–2	22 (57.9%)	23 (50.0%)	0.163
	3	10 (26.3%)	20 (43.5%)	
Subtypes	Non-TNBC	25 (65.8%)	29 (63.0%)	0.974
	TNBC	13 (34.2%)	17 (37.0%)	
cT stages	1–2	27 (71.1%)	28 (60.9%)	0.916
	3–4	11 (28.9%)	18 (39.1%)	
cN stages	2b or 3b	27 (71.1%)	36 (78.3%)	0.613
	3c	11 (28.9%)	10 (21.7%)	
FNABx for IMN	(−) or unknown	16 (42.1%)	28 (60.9%)	0.135
	(+)	22 (57.9%)	18 (39.1%)	
Extent of the IMN	Single ICS	18 (47.4%)	19 (41.3%)	0.737
	Multiple ICS	20 (52.6%)	27 (58.7%)	
IMN long diameter	< 1.0 cm	20 (52.6%)	22 (47.8%)	0.826
	≥ 1.0 cm	18 (47.4%)	24 (52.2%)	
Neoadjuvant chemotherapy	Not done	7 (18.4%)	11 (23.9%)	0.731
	Done	31 (81.6%)	35 (76.1%)	
Primary surgery	BCS	20 (52.6%)	20 (43.5%)	0.538
	Mastectomy	18 (47.4%)	26 (56.5%)	
Axillary surgery	ALND	32 (84.2%)	42 (91.3%)	0.509
	SLNB	6 (15.8%)	4 (8.7%)	
RT dose to IMN ^2)^	50–63.5 Gy	15 (39.5%)	20 (43.5%)	0.882
	63.6–70.4 Gy	23 (60.5%)	26 (56.5%)	

^1)^By the Mann-Whitney U-test

^2)^Radiotherapy dose was calculated using the biologically equivalent dose in 2 Gy fractions (EQD2) assuming the α/β ratio of 3.5 Gy

Abbreviations: *mo* Months, *TNBC* Triple-negative breast cancer, *IMN* Internal mammary node, *FNABx* Fine needle aspiration biopsy, *ICS* Intercostal space, *BCS* Breast conserving surgery, *ALND* Axillary lymph node dissection, *SLNB* Sentinel lymph node biopsy

This study has some limitations. The follow-up duration of our patients was short. With a median follow-up time of 58 months, the rate of treatment-related toxicity might be underestimated. As the likelihood of radiation-related toxicity, such as cardiac disorder, increases with time after RT, a longer follow-up is necessary to ascertain the incidence of treatment-related adverse events [33, 34]. Moreover, we could not assess the prognostic significance of some variables. Among the patients receiving NAC, information about the histologic grade of the breast tumor and the number of positive ALN was not available when a pathologic complete response was obtained. Furthermore, we could not evaluate the prognostic impact of the NAC response on the outcomes because many of the patients in our study did not receive NAC. To assess prognostic significance of the above-mentioned variables, we have a plan to conduct a multicenter retrospective study including a large number of patients. Despite the limitations, the findings of our study have important implications for determining the optimal radiation dose for the management of cIMN+ breast cancer.

## Conclusions

Patients with cIMN+ breast cancer achieved favorable outcomes after the combined treatment of breast surgery, IMN-targeted RT, and systemic therapy. In patients with IMN size ≥1.0 cm, a high IMN radiation dose was significantly associated with improved DFS. Therefore, it might be necessary to administer an EQD2 > 63.5 Gy to the IMN to achieve favorable outcomes in patients with large IMN metastasis from breast cancer.

## Data Availability

The datasets used and/or analyzed during the current study are available from the corresponding author on reasonable request.

## Abbreviations

3D-CRT: Three-dimensional conformal radiotherapy
ALN: Axillary lymph node
BCS: Breast conserving surgery
cIMN+: Clinical metastasis to the internal mammary lymph node
CT: computed tomography
CTCAE: Common terminology criteria for adverse events
DFS: Disease-free survival
EQD2: Biologically equivalent dose in 2 Gy fractions
ER: Estrogen receptor
FNABx: Fine needle aspiration biopsy
HER2: Human epidermal growth factor receptor type 2
IHC: Immunohistochemistry
IMN: Internal mammary lymph node
LRRFS: Loco-regional recurrence-free survival
MRI: Magnetic resonance imaging
OS: Overall survival
PET-CT: Positron emission tomography-computed tomography
PFS: Progression-free survival
PR: Progesterone receptor
RT: Radiotherapy
SCN: Supraclavicular lymph node
TNBC: Triple-negative breast cancer
US: Ultrasonography
